# Supplementary material for: A DNA Replication Mechanism Can Explain Structural Variation at the Pigeon Recessive Red Locus
Source: Biomolecules. 2022 Oct 18;12(10):1509. doi: 10.3390/biom12101509 (PMC9599118; doi:10.3390/biom12101509)
Supplement: Supplementary file 1 [file biomolecules-12-01509-s001.zip › Supplemental Figure S1.pdf]

Figure S1. E^{+} allele annotated relative to e^{1}

Annotation Key

Same orientation in e^{1}
Inversion 1 in e^{1}
Inversion 2 in e^{1}
Inversion 3 in e^{1}
Sequence that is duplicated between inversions 2 and 3
Deleted in e1
| insertion in e^{1}
SINE/MIR
simple repeats
low complexity GA rich
enhancer MCS4
enhancer MCS5
enhancer MCS7
Sanger-sequenced gap in reference genome

GTGACCCAAAGCACTGTTGTCAGCCCTACCATATGGACTATGAGGTCTTCAGAACAACTCTCTGATCAGCCCTAGGTGCGTACATGGCTGCAGCACTGAC
TAAGAGCTGAGCTCAGCAGCCTGACAGTGAGTGCAGGTTGGGCAGACGGTGACCTGCCTGAAATGTGAACAGAGCTGGTCTGGAGCACACAAATTGCTTC
TGTTGGCCAAAACACATGTCACCAAAGCATTTCCTCCTCCCCACACACACCAGGGTGGTGCAAAAACAGGCTTGAGCACTATCTCTGTACCTTAGCATCC
TCTGAGAGATGCGCGATGCCACCAGCATGGACTCCTCCACCCCCTCCCCGCTCCCTGTACCACCACAATGAGCATGCCTTGTCGTAGAGGGTTAATAAAT
GATGCAAGAAAGGAGCCTTTTAATGCATGGTGACCTTTGACCTTTCATAAATCACAGTGTTGGGCTGTGAGTGCGCGGGGGGCGCAAGGCAGTGGGGGA
GAGGAATGGGAGGGGGTCCATGCAGCTGCGCCACCGAGTTGACATTGTTCCCACCATCACTAAAGTGCAACAAATCCCTCTATTGTGTTCCTGGTTTATC
TGGTTCCTCTTGTTTATTAGCAGAGGTTGTTTGGCGCTGGCTCCTGAGCCCTGGGCGGGTGGAAAGAGTGTTGGCACGCACCAGAGGAGGAGAGGGAGGG
AGTGGGGGGGACCGGGACGGGAGGACGAGAGCAGTTCATCAATGGCTGTATTGTCCCATCTTTTTTGTTGCTCCTGTAATGATATGTTCGAAAAAAGGGGAT
TAAAAAAAAAAAAAGGCAGGGGGAGACAAGAGAGGGAGAGAAAGACAAACCACATAGAAGGACTTGGTAAGAATGGCCGGTCTGGGCCCGCAGCGGATGC
TGTCGCAGCTGGGGAGGATGGGTCGGGACGGAGCCGAGACGGGGCTGCTCTGTGCCTTTATGCTGCTATTCAGGTCCAGAGTCGTGAGCCCCAGTGGCTC
AGTCAGGCTGGGCCCACCCGTGCCAACTCTATTAAATGACCAGTGGGGAMCATGTGCCCATGGTGGAGAGGATGGGGTGGCAGGAGCGAAGGGCTKGGGC
TGGTGGGGAMGGGAGAGGAGGGCAATTCTTCACATCCATCAAGTCTGAACCTGCCTTTGTACAGAAGAGGCAGTTGGGGGGCACAGCACCTTCTCAAAGC
ACTGGCGGTGGACCAGATGATCCCTGTGGTCCCTTTCCAATCTGGTATGCTATGCTTCTATGATTCATCCCTCTTGGTCATTTTTCCCCAACCTGCTCCC
TCATTATGAGAAGCAACAGGGTCCCTTCCTAAATTAGCAGAGATTTTCTTGTGCCTGGACCTTGCTTACACGAGCATTTTTCCCCCTGGAGCTGCTGACT
GCCCATGGAGAG|CTCCTCTGGTTTTCATAGGGATCGGCAGGACAGTGCTGGGAAATGATGGCACTTTGGTTGCCGCTTTACCCAAAGCTAGATGATGATG
TGAATAAAACACCAGGGCCACAGGGACTTTGTTCATCATTGCTCTGCCTTAGCTACCCATGGAGCTGGACTGAAGCAAGCATCCTAAGGGGTGCACGTGC
CACCATAAATTGCCTTGTTGAGATCAAAGTGAGATTTATTGAGAGAAGAGTGAAATTCGGTATGTGATGTTCAAGGGCATTTTCTAGTTCATCTCAGCCC
ACCATATAGCTGTTCTTTTCCCAACCACACCAGCCGTTTCTGCTCCAGTGTCTCCATTACCTCAGAGGTGTCGCATAGGGGCACATTGATCTGGACATGC
ACAGGTGCTCTGCTGTGATTTATTTTTTTTCTCCAAATGGTATAATTCAGAGATTAAAACATTCTGCTGAACTTGAAATAGCAGGCTTTATTTTGTGGTT
ATTAGGCTCTTGTGCCTGACCTCTCATCTTTCAACCTGGGTCAAGTTTTAAATGCAAAATGCTGGTTTGAAATGAAATGTCAGAAATTCCTTTGAAAATG
TCCATTCGCAACATTTTGGCATCTAGAAAACAGAATAATTCAGGAAGATTGCATTGAAATGAAATGTTTTTGATATTTTAAAAATCCCCCTCCCACACTC
ACTTTCCAGCCAGAATTTCTTGTTGAATTAGGTCTGGATCTGCAACTGGTTTCCTTCCCCTAAGTCCCATTTCTTGGCAAATTATTTACCAATAAATGCT
TCCCTAGTACCAGTCTTTCCTACCCTCAGATGATGCCACATCCTTGCTATCCCTGAGAGGTCTCAGGGTCTCCACAAAGCACTGGAGTGTGTAGGAGAAG
TGGAGAGGAGGGGATGGGACACACTCTCCAGTATTGCACAGCAATGCACGCGGTGAGACCCTTTTTCACCTGAGTGGAAAATTAAGTGTGTCAAAGCTGA
GAACTATTGCTGACTTGATGAAAGCTAACATTTTTCGATACCCTCTTGTCCCATGTAAACATAAAATGAGTCAGCCATCCCAGTGGCCACATAGAGATGA
GCCAGATAGTGACAACAGGATTATACAGCTGTCAACAGCTTCCCAACCCCACTTTCAAGAGTGGTTCAGCTCCCCACCTTCTCCTCTGCCCATCACACTC
CTTCTCCAATACAAGCCAGATCAGCGTCTCCACGATGGAAAATTCTCAAACCTAATTCTGGCTTTCCCCAGTCAGATAATGCTACATATCTAGCAGGTAC
CTCTATACCCAGTGTCATCCTAAGCATATTCACGTAACCTCTGAAAAGAAGGAATGGATAACTCCTGTCACCAGTTGGTATAACTACTTGTTCAGTTAAA
TCATCCAGGTCCACCTGTCCTTCCCTTCCTCCAGTCCAGTGAGAAGGGACTTCATAGGACTTCTGGGTTATGAACCTGAAGAGTTATAGGGTTTTGCTTG
GAAAAATCCGGTCACAAAATTAGTTTAGCAGCAACCTGGAAACAAAGTACAGCTTGCCTGTGCCAAGTGGAACCAGTAGCAACATCAACCCTACACCTGA
GCTGGACAGTCACAGGAATATATCAAAGCCAAATCCCCTTCTGAGACTCTCATGTCCTTCTTCTTTCTTGTGGGCAGTCTAAGCTTTGCCTAACCTAAGG
TCTGCCTACCAGTCTCATCAGCCCTCGCAGCAGGTGGCACTGGTGGCTTTGAAGGAGATGCTCTTGTCTGCCCAGMGTAGGTCATGTCACATCACAGAAA
GGACAGAGCGCTGGGACCTGGATTTATAACACACAAGTGCATCCCTGTGTGTCCTTAAAGCAAAGAGTAGCTTGAGCTGCTGTCCCTGAGAGCAGCATCT
GGATCTCACCACAGATATCCCCAAGAGCATTCGGTGGGTTGTAATGAGGACCAGCCAGGTTGCCTCAAAGCTGTGCGTGCACAGCACTGTATTTTGCAGC
AGGGAGCACCTCTCCTTCCACATGGAGGAGAAGAGGAAGAGAAAATGGAAGAGAAAATAAATTATCATCATGCACATGAAGAGGACATCTCATCCCAGAC
AGCATGGGAGACGCTCCTTGGACACTGCGGTCTCAGCTGTGCTCTTGGGGGCCTGACTCTGCTGCTTCCAGTGGCATCCCTTGGGACAAGACTCTGGTAC
TCACCGTGAACTTGACAGTCTGAGCAGCTGTCAATAATCTTACYGTTAGGTTTTGCTTTCCAGATCCCAGGGAAGTTCAAGGAAGCACTTGTCTGCCAGC

AGTGGACTGGGAGTTCTTAAATGAGTATCAGCATTAAGCTGGAAATTCACAACAAGCCTGGATCAAGTTCCTAATCAAGAGGCACATTTGGGACATTTTA
AACCCAAATGCCACCTCTTGGGAGCAGCACAGGCCCTGCACCTCTGCAGAGAGTCACACACTTTGCTCAGGAGCAGGAGAGGAGGCACAGCCCGTCACCC
CCTGGGACACKCAACACGGTGACTGGAGGAGCATGGCACTGCTGGCGGAGTGTCCCAACCCAGATGGCCCAGCTGAACCTGCTGGGCAGCAGAACTCCCC
GGCACTGCCAACTGCACCAGCACCTGCTCCCGTGCTGCCCGAAGCACAGCTGTCTCCTGTCCCCTCTTCTCCTTACTCCCCCAGGAGATTGCACAGCATC
CGTGGTCCCCTGGCCACCAATGCGGCCCTCCCGCCAGGGGTCCCCTCGCCCACTGGCCAGGTCTGTTTGAATCGAGGCATGAGAATTTCTCCCCCATTCG
CGCAATCCTCTTGCCTCCCCCCAGCCATCAAAATCATCCTTCCCTGCCTCCCTCTTTCTCCTTCCCGCCGCCTCCCCCTTCTCTCCCTGGAACTTCCCTG
CAGCTGCAGAGCTGGCCAGACAATCGGGTGTTTTAGAGAAATACATTTTTGGGCTAATTTGCCATGCATAAGTGTTTCTCTTACCCTTTAAAGGCAGGGA
ACAAAGAGCACATTCTCCGGGAGCTCTGCCCTGTTGTGAGCAGCCCTGCAGCTGTACAGCGCTGTGCTACCCTCCGCGCTCCTGCTCCGCTCTCTGCTAG
CTGCACAAGGAGACAGGGCTGAGCTTCTCCCCGAGACAACGCCATCTCCTACCCATCACTACACATTTCTTCCTGGTTTGTTTTGTTTTGTTTTGTTTTT
AAAGTAGAGCCAGAACAGTGGTGAGGGAAGGGATTTGGCAGCACTGTCACCTAGACTGACTTGCCAGATCATCCAGGTACAGTCTTTTGTATTGAGACAT
CATCTATTTGGAAAGTCTGAATTTTTCTTTATTTGGGGCTTAATCCTGTGTCCGGGTTAAGTATTCTATTCCCTCAATAAGTAACACTGTGGCAATGCAT
TACACACACCCACATTAGACTGCATGAAATTTTGTGCCATGAAGGAGCCCTAGTCATTAAAAAAGAAGTCGTGTGCGGGGTTTTCAGGTCCCGTTTCCTC
ACTTAAAGCACTGGTAGTTATCCCTGATTGGGGAATTTCTCAAACACGAGGATTTTCCTTTGTAAGGATAGGATGTTGAATGATGAACAGATTTGACCTG
GTGGACCAGATCAGGAATGAGATACAGATATTCTGGCATGAACAACTCATGAACATGCAAGGTTGTGGGAGAAGATGGCCTGGAGCAGCTGGACACTGAG
GAACTGAAGTTTTTTATCAGCTTTTTGGGGACCTCATTTCAGCACTTGCTGAATAATCTTTTTCCTACTCATGGAAAGCTGGTCCAGCAAATGGGGGCAA
CAAGTTCTGGTGTAAGTGAAACAAGGCTTTTAAAGAAGCCGCAATGAAGTCATTCACCCCATTTCAGCAGGCAGGTCCCACCAAACTCTTCCCACCAGCC
CAATCCCATCCACTGTAAATAATCTTAACTCAGTCCAAACCAGCCACATTTTCAGCTCTGAATTCAACACAATCTAAAGAAGCTAGCCCAGTCAAGTCTA
GCCTATCCAGAGCATGCCTATCACAAGCTACCATCTCAAACATGCATTCAGATTGCACTCAGTCCATGTAAGTGTAGCTCATTTGGACTGAGGCTAGTGT
AACTCAGCCCAACCCAGCCATACCAGGACATTCTGAGACATTTCAAGCCACGCTCCAGTTGGGGACTAGTATAGTCTAGCCTGTCAACTTACACTCAAAG
TCTGACCCAGTAACGTGATGAACCAGGATGTGCCAGCTGCAAGGCAACAGAAGCTCAGTTTTTACACACTTGAAATGTGTTTTTAGGTTTGAAAATGTCT
TACCCAGAGTACAGAGATCAGCATGGCTTTCAGAGTTCCAGCTTGTGGGACCTTGTCTCAGATAGGTTAACAGCATTGGTCCATTTGAAATGCTCTTCAC
ATCATACTGATACAAATACACTTGTTCTTCAGTTCTCTCCAGCACATTGCACCCTATAATTTTGCACCCAGAAGATCCTCTTGACCCAAATCCAGATGGA
TCTCTTGCAAATACAGAGAAAGATAGAGACTTTGTTTTAAAATTCTTCTATCTAAGAAGAGAAGGGAAAGGAGGAGAGGAGAGGAGAGGAGAGGAGAGGA
GAGGAGAGAGGAGAGGAGAGGAGAGGAGAGGAGAGGAGGAGGACTGCAGTCAGCAGAAATGTATTTGGAGAAGCACATAAAGCAGGAAAAGGAGAAGGTC
TCACGAACTGAAGAGCAAAATCCTAAGCCTCTCAGCTAGGAAAGGCCAGCTTGGAGGAAACAATAGCTGGAGGAATGTACAGTAGAAGATGGTGTAATTG
GCAACAGCACCCCAATGCTAGGACTGTGGGGCAGGTTGAGCCTCACCTGGCACAGCCCACAAAACCCTTCAGTTCCTTTCTGGCTTTTTGAGCTCTTTGC
ACTCAGTTCTTCAGTAGCCATACACATGTAATAACTTGGTTACAGTAGGAAGCCATTAGTGGAGCTGAGGCTTAAAGACGTGTGTTTATTGACAGTGTGG
TGGCACTTGTTCCACACAGAGCACCACCAGCTCTTAGCTCAGCTGTGGTCTTACTGCTCTGAGACTCCTGCTTTGCTATGCTACTGCGAATGAGTTGTAT
GAGGAAGCAGTGGAGAAATTCAAGATTCTTAGTTTCCAAGCTGCATTTTAAACTCCAGAATTCACTCTTTTGACCTTACAAGTGACTCCAGTAAGCGCCA
GTCAAGTATTAAAACCGTAATGCCAAGAAAAAGTCAATTTCTCCTGGACTCAGAGCTGCAATAGTGAAAATCCCATTGAGCAATGGGTTTAATAATATTT
AGCATCCCCTGTGGGTAAATGTTCTTCGATCCCTCTAGGAGACTGGACTCGAACATTTCCCTGGCAGGGCTGTAGGGGACATCACACAGCACAGGTGGTC
TGAGTGGCACAGGTAGGTTGGACGCCAAGACCAAGTGCCAACACAAACACTCTGAAGCCCAGGATGAGGTTAGCAGGACCAAAATCAGCTCCAGCCTTTG
TGGTTACCATCTCAGTAAGATAGAAAGAGAGATAGAAAGGAGACAGAAGGGAGTGAGAAGGTTATGGAGTGGGGGAGGGCTGGGAGCCTGCAGACTTCTG
TTGCCACATGATTTTCCTGATGTTTTCAAAGTGCTGCTCTAACACACGCGCATGAGCCCCATGTGACAGCTTATTTACGAAAGCACAGGCGTAATGTTGG
GTACATCTCGCATCCGCTCAGCAGCCCACCTGACTGCCAGCTCTGTGTGCACCCACTGTGTCCTCCCCATCTGCGCCATCGAGCCAAGGGGGATGGGTGA
CCCATGATGGGTGGACATTGCTCCTGTCTTGGCAGGGKGGGGAAGCGAAGAGAAGGACTGTCTTTCCATTTCCAAACTGCTCTTTACTTCTCTTTGCCTG
GCACTACATGAGGAGACTCCAGACGCAGGATGCTGTCCCTTCTGTAAGAGAAAAAGCTTTTATTTCCGATCTGGCTGTCTCACTTCTTTTAATTTGCAGA
GCACCTGGAAGCAAGC**TCAGATACAAACTGATTCAGCGTATGCCAATATCCCATACTTGATCTTCAAACAGATGCAGATTCTCTTTCCTTGGATATAAAT**
TTTTCTTGTACCATCATCACAGAAGTTCCATTTCACCCAAAGATCCACTCAACAAAAGAGGAAGCCTCCTTAGAAAGGTCAGGCAGAAGGCTAAGGGAGA
GAGTTCACAATGAAATAAAGCTGGTTGTTTCTTCAGGGATGGAGGATTTGGCCTACCTTCAATTCATTTGCATACAGAACCCCTAGCTCCACTAAAATTG
TCCATCTCTGCCAAAAGGAGGTCTAACACATGAGCAAGAGAGATGCAGTCTGCGAAAATGGAGTTTCTGAAGGACAGCAATAGGAACTGAGTTCACAGAG
GCTGTTAKGGGACAAGATTATGCTGCCATAAAGCATATATTTCAGGAATGGCAACTTCCTTTCCTTTCTCTCCCCATAGTCCTAGGTGCTAGCATTCCTA
GAAAAAATGTCAGTGGAAAAAAGTCATCACCTCATATGCTGAAAAAAAAACACAACAGTGACCTTTGCCAAAACAGAAGCTCCTATGAAGAAAGGATATC
TGACATTTTAAAACTTTGATATGGTCAAACCACACAATGTCATCCCTACCTCCTCTTTCCAGACAATCAGTCCTTGCTTTTAATGACCCAAGAGCCAAGG
GCGCTACCTCACCACACAGATCACGCCTCCCATGGCCATGCCTCTTTCCTGCACCTCAGTAAGTTGCTTCTAGGAAGGGTTAATTCAACCAGGCCTTTT
ACTCCAAGTTGTTTTCCAAGTAGCTGAGAGAATAGGTCTCTTGTTACAAACATGGCTTCTGGGCATTTGGTGATGCTTTCCCTTTTCTCTCTCTCTGTAC
CACAAAAGAACCTTTTGTTCTTGTTCCTCTGTCTCATTTACTCTCCCCCAGCCTGCTCTCTCCTCTTCCCTCCATTGGCTGCCAAGKGACTAAGGAATGG
CAAAAAATGACCATGGCGCGGTCATGGCAAATATTTCTTCAGCT|ATTATCTGATAGAGACCTTGAGCATCTACGCGAAAGGTCCTCTGCCTTCCCTCAGG
CTTCTCCTAGAGATGGGGAAAAATGAGTCAGACACAACATGGCAGGATATAAGTTGAATCAGCCCAACATGCTAATGGGAATGTCTTGCGAATGGAGTCA
TGGTGCCTGTCTCAGGGAAATCAAATGATAGGGACCCAGAACTGGCCGGAGTCCAGTGGCAGATTCAATGGGTCTAGATTTTTATAGGAACTCTCAATTT
ATTAAAAAGCACACAGTAGGCACGAAAAGTCATTTTCTTGCCTCCACATTTACTGATTCAGCTGTTTCTAAGCTGGAGCTGACACTCTTTGTCAGATGGG
TTGTAATATTGTATAGTTTCTCCACACCATTTATGTAAATAAATTGTGCATGATCACCCACCACAGCAAAGCTGTGGCAAATCCCCATGGATATCACGTC
TTCCCACACCCCACTGAAATTACACCTCAGGCATTCCCACATACATACTTCTAGCACGTGCGGCTTCACTGAGTCATTTTACACGTACATACATAGATAC
ACAAAAGTTCACATGACGGTAAAGTCACCGAAACTTCCTTGGTGACCTTTCTGCAACAGAAACAATTCCCCAGACAAATTCCCACCTCCCTACCTTTTAG
AGCATATGGGTATATCCTTCACTGATACTGCCTTCTGAAAAAAAAAAAACAAACTAAAATTCTTCAGTCTCACCTGAAGCAAATCTTGCTGTTTCTGCCT

TCTCATCTTCTTCTTTACTGACCCTCTTCACTCCTCTGACTACCCTTTGTCCCGAGTCCATCTCCGTCCATGTTGTGCTTTAGGTGAACATGGCATTTGA
CGGCTGGGTCCATCTGCCAAGCTTTTTGCACTCTTCTCCCATTCAGTGCCTTAGCTATTTTGGAAGGCCCTTGAAAAAATCATTAAATTTCAGCCTTAGT
GACTTCTCGCACACACGCATGCAAAGCTGTGGATATAAGGCGTTATCTGGAGAATTTTTATTCCTGTAGAGAAGGGGAATGGACTATACAGATAAAAGGG
CCATTATATTCCCACATTAGAAGGACTTCTTGTCTGGTGGAT
