# Supplementary material for: A DNA Replication Mechanism Can Explain Structural Variation at the Pigeon Recessive Red Locus
Source: Biomolecules. 2022 Oct 18;12(10):1509. doi: 10.3390/biom12101509 (PMC9599118; doi:10.3390/biom12101509)
Supplement: Supplementary file 1 [file biomolecules-12-01509-s001.zip › Supplemental Figure S2.pdf]

Figure S2. e^1 allele annotated relative to E^+

Annotation Key
In same orientation as E+
Inversion 1
Inversion 2
Inversion 3
Duplication relative to E+
Insertions relative to E+
\ one letter deletion relative to E+
SINE/MIR
enhancer MCS4
Sanger-sequenced gap in reference genome

GTGACCCAAAGCACTGTTGTCAGCCCTACCATATGGACTATGAGGTCTTCAGAACAACTCTCTGATCAGCCCTAGGTGCGTACATGGCTGCAGCACTGAC
TAAGAGCTGAGCTCAGCAGCCTGACAGTGAGTGCAGGTTGGGCAGACGGTGACCTGCCTGAAATGTGAACAGAGCTGGTCTGGAGCACACAAATTGCTTC
TGTTGGCCAAAACACATGTCACCAAAGCATTTCCTCCTCCCCACACACACCAGGGTGGTGCAAAAACAGGCTTGAGCACTATCTCTGTACCTTAGCATCC
TCTGAGAGATGCGCGATGCCACCAGCATGGACTCCTCCACCCCCTCCCCGCTCCCTGTACCACCACAATGAGCATGCCTTGTCGTAGAGGGTTAATAAAT
GATGCAAGAAAGGAGCCTTTTAATGCATGGTGACCTTTGACCTTTCATAAATCACAGTGTTGGGCTGTGAGTGCGCGGGGGGCGCAAGGCAGTGGGGGGA
GAGGAATGGGAGGGGGTCCATGCAGCTGCGCCACCGAGTTGACATTGTTCCCACCATCACTAAAGTGCAACAAATCCCTCTATTGTGTTCCTGGTTTATC
TGGTTCCTCTTGTTTATTAGCAGAGGTTGTTTGGCGCTGGCTCCTGAGCCCTGGGCGGGTGGAAAGAGTGTTGGCACGCACCAGAGGAGGAGAGGGAGGG
AGTGGGGGGGACCGGGACGGGAGGACGAGAGCAGTTCATCAATGGCTGTATTGTCCCATCTTTTTTGTTGCTCCTGTAATGATATGTTCGAAAAAAGGGGAT
TAAAAAAAAAAAAAGGCAGGGGGAGACAAGAGAGGGAGAGAAAGACAAACCACATAGAAGGACTTGGTAAGAATGGCCGGTCTGGGCCCGCAGCGGATGC
TGTCGCAGCTGGGGAGGATGGGTCGGGACGGAGCCGAGACGGGGCTGCTCTGTGCCTTTATGCTGCTTATTCAGGTCCAGAGTCGTGAGCCAGTGGCTCA
GTCAGGCTGGGCCCACCCGTGCCAACTCTATTAATGACCAGTGGGGACCATGTGCCCATGGTGGAGAGGATGGGGTGGCAGGAGCGAAGGGCTGGGGCTG
GTGGGGAAGGGAGAGGAGGGCAATTCTTCACATCCATCAAGTATGAACCTGCCTTTGTACAGAAGAGGCAGTTGGGGGGCACAGCACCTTCTCAAAGCAC
TGGCGKTGGACCAAGATGATCCCTGTGGTCCCTTCCAATCTGGTATGCTATGCTTCTATGATTCATCCCTCTTGGTCATTTTTCCCCAACCTGCTCCCTC
ATTATGAGAAGCAACAGGGTCCCTTCCTAAATTAGCAGAGATTTTCTTGTGCCTGGACCTTGCTTACACGAGCATTTTTCCCCCTGGAGCTGCTGACTGC
CCATGGAGAGTCACTGAGTGCAATCTGAATGCATGTTTGAGATGGTAGCTTGTGATAGGCATGCTCTGGATAGGCTAGACTTGACTGGGCTAGCTTCTTT
AGATTGTGTTGAATTCAGAGCTGAAAATGTGGCTGGTTTGGACTGAGTTAAGATTATTTACAGTGGATGGGATTGGGCTGGTGGGAAGAGTTTGGTGGGA
CCTGCCTGCTGAAATGGGGTGAATGACTTCATTGCGGCTTCTTTAAAAGCCTTGTTTCACTTACACCAGAACTTGTTGCCCCCATTTGCTGGACCAGCTT
TCCATGAGTAGGAAAAAGATTATTCAGCAAGTGCTGAAATGAGGTCCCCAAAAAGCTGATAAAAAACTTCAGTTCCTCAGTGTCCAGCTGCTCCAGGCCA
TCTTCTCCCACAACCTTGCATGTTCATGAGTTGTT**CAGCTTTATTTCATTGTGAACTCTCTCCCTTAGCCTTCTGCCTGACCTTTCTAAGGAGGCTTCCT**
CTTTTGTTGAGTGGATCTTTGGGTGAAATGGAACTTCTGTGATGATGGTACAAGAAAAATTTATATCCAAGGAAAGAGAATCTGCATCTGTTTGAAGAT
CAAGTATGGGATATTGGCATACGCTGAATCAGTTTGTATCTGAGAAACAACCAGCTTTATTTCATTGTGAACTCTCTCCCTTAGCCTTCTGCCT
GACCTTTCTAAGGTCATTATCTGATAGAGACCTTGAGCATCTACGCGAAAGGTCCTCTGCCTTCCCTCAGGCTTCTCCTAGAGATGGGGAAAAATGAGTC
AGACACAACATGGCAGGATATAAGTGAATCAGCCCAACATGCTAATGGGAATGTCTTGCGAATGGAGTCATGGTGCCTGTCTCAGGGAAATCAAATGATA
GGGACCCAGAACTGGCCGGAGTCCAGTGGCAGATTCAATGGGTCTAGATTTTTATAGGAACTCTCAATTTATTAAAAAGCACACAGTAGGCACGAAAAGT
CATTTTCTTGCCTCCACATTTACTGATTCAGCTGTTTCTAAGCTGGAGCTGACACTCTTTGTCAGATGGGTTGTAATATTGTATAGTTTCTCCACACCAT
TTATGTAAATAAATTGTGCATGATCACCCACCACAGCAAAGCTGTGGCAAATCCCCATGGATATCACGTCTTCCCACACCCCACTGAAATTACACCTCAG
GCATTCCCACATACATACTTCTAGCACGTGCGGCTTCACTGAGTCATTTTACACGTACATACATAGATACACAAAAGTTCACATGACGGTAAAGTCACCG
AAACTTCCTTGGTGACCTTTCTGCAACAGAAACAATTCCCCAGACAAATTCCCACCTCCCTACCTTTTAGAGCATATGGGTATATCCTTCACTGATACTG
CCTTCTGAAAAAAAAAAAACAAACTAAAATTCTTCAGTCTCACCTGAAGCAAATCTTGCTGTTTCTGCCTTCTCATCTTCTTCTTTACTGACCCTCTTCA
CTCCTCTGACTACCCTTTGTCCCGAGTCCATCTCCGTCCATGTTGTGCTTTAGGTGAACATGGCATTTGACGGCTGGGTCCATCTGCCAAGCTTTTTGCA
CTCTTCTCCCATTCAGTGCCTTAGCTATTTTGGAAGGCCCTTGAAAAAATCATTAAATTTCAGCCTTAGTGACTTCTCGCACACACGCATGCAAAGCTGT
GGATATAAGGCGTTATCTGGAGAATTTTTATTCCTGTAGAGAAGGGGAATGGACTATACAGATAAAAGGGCCATTATATTCCCACATTAGAAGGACTTCT
TGTCTGGTGGAT
