# Supplementary material for: A DNA Replication Mechanism Can Explain Structural Variation at the Pigeon Recessive Red Locus
Source: Biomolecules. 2022 Oct 18;12(10):1509. doi: 10.3390/biom12101509 (PMC9599118; doi:10.3390/biom12101509)
Supplement: Supplementary file 1 [file biomolecules-12-01509-s001.zip › Supplemental Figure S3.pdf]

Figure S3. *E⁺* allele annotated relative to *e²*

<u>**Annotation Key**</u>
Same orientation as *e²*
Deletion in *e²*
SINE/MIR
<u>simple repeats</u>
low complexity GA rich
enhancer MCS4
enhancer MCS5
enhancer MCS7
Sanger-sequenced gap in reference genome

GTGACCCAAAGCACTGTTGTCAGCCCTACCATATGGACTATGAGGTCTTCAGAACAACTCTCTGATCAGCCCTAGGTGCGTACATGGCTGCAGCACTGAC
TAAGAGCTGAGCTCAGCAGCCTGACAGTGAGTGCAGGTTGGGCAGACGGTGACCTGCCTGAAATGTGAACAGAGCTGGTCTGGAGCACACAAATTGCTTC
TGTTGGCCAAAACACATGTCACCAAAGCATTTCCTCCTCCCCACACACACCAGGGTGGTGCAAAAACAGGCTTGAGCACTATCTCTGTACCTTAGCATCC
TCTGAGAGATGCGCGATGCCACCAGCATGGACTCCTCCACCCCCTCCCCGCTCCCTGTACCACCACAATGAGCATGCCTTGTCGTAGAGGGTTAATAAAT
GATGCAAGAAAGGAGCCTTTTAATGCATGGTGACCTTTGACCTTTCATAAATCACAGTGTTGGGCTGTGAGTGCGCGGGGGGCGCAAGGCAGTGGGGGA
GAGGAATGGGAGGGGGTCCATGCAGCTGCGCCACCGAGTTGACATTGTTCCCACCATCACTAAAGTGCAACAAATCCCTCTATTGTGTTCCTGGTTTATC
TGGTTCCTCTTGTTTATTAGCAGAGGTTGTTTGGCGCTGGCTCCTGAGCCCTGGGCGGGTGGAAAGAGTGTTGGCACGCACCAGAGGAGGAGAGGGAGGG
AGTGGGGGGGACCGGGACGGGAGGACGAGAGCAGTTCATCAATGGCTGTATTGTCCCATCTTTTTTGTTGCTCCTGTAATGATATGTTCGAAAAAAGGGGAT
TAAAAAAAAAAAAAGGCAGGGGGAGACAAGAGAGGGAGAGAAAGACAAACCACATAGAAGGACTTGGTAAGAATGGCCGGTCTGGGCCCGCAGCGGATGC
TGTCGCAGCTGGGGAGGATGGGTCGGGACGGAGCCGAGACGGGGCTGCTCTGTGCCTTTATGCTGCTATTCAGGTCCAGAGTCGTGAGCCCCAGTGGCTC
AGTCAGGCTGGGCCCACCCGTGCCAACTCTATTAAATGACCAGTGGGGAMCATGTGCCCATGGTGGAGAGGATGGGGTGGCAGGAGCGAAGGGCTKGGGC
TGGTGGGGAMGGGAGAGGAGGGCAATTCTTCACATCCATCAAGTCTGAACCTGCCTTTGTACAGAAGAGGCAGTTGGGGGGCACAGCACCTTCTCAAAGC
ACTGGCGGTGGACCAGATGATCCCTGTGGTCCCTTTCCAATCTGGTATGCTATGCTTCTATGATTCATCCCTCTTGGTCATTTTTCCCCAACCTGCTCCC
TCATTATGAGAAGCAACAGGGTCCCTTCCTAAATTAGCAGAGATTTTCTTGTGCCTGGACCTTGCTTACACGAGCATTTTTCCCCCTGGAGCTGCTGACT
GCCCATGGAGAGCTCCTCTGGTTTTCATAGGGATCGGCAGGACAGTGCTGGGAAATGATGGCACTTTGGTTGCCGCTTTACCCAAAGCTAGATGATGATG
TGAATAAAACACCAGGGCCACAGGGACTTTGTTCATCATTGCTCTGCCTTAGCTACCCATGGAGCTGGACTGAAGCAAGCATCCTAAGGGGTGCACGTGC
CACCATAAATTGCCTTGTTGAGATCAAAGTGAGATTTATTGAGAGAAGAGTGAAATTCGGTATGTGATGTTCAAGGGCATTTTCTAGTTCATCTCAGCCC
ACCATATAGCTGTTCTTTTCCCAACCACACCAGCCGTTTCTGCTCCAGTGTCTCCATTACCTCAGAGGTGTCGCATAGGGGCACATTGATCTGGACATGC
ACAGGTGCTCTGCTGTGATTTATTTTTTTTCTCCAAATGGTATAATTCAGAGATTAAAACATTCTGCTGAACTTGAAATAGCAGGCTTTATTTTGTGGTT
ATTAGGCTCTTGTGCCTGACCTCTCATCTTTCAACCTGGGTCAAGTTTTAAATGCAAAATGCTGGTTTGAAATGAAATGTCAGAAATTCCTTTGAAAATG
TCCATTCGCAACATTTTGGCATCTAGAAAACAGAATAATTCAGGAAGATTGCATTGAAATGAAATGTTTTTGATATTTTAAAAATCCCCCTCCCACACTC
ACTTTCCAGCCAGAATTTCTTGTTGAATTAGGTCTGGATCTGCAACTGGTTTCCTTCCCCTAAGTCCCATTTCTTGGCAAATTATTTACCAATAAATGCT
TCCCTAGTACCAGTCTTTCCTACCCTCAGATGATGCCACATCCTTGCTATCCCTGAGAGGTCTCAGGGTCTCCACAAAGCACTGGAGTGTGTAGGAGAAG
TGGAGAGGAGGGGATGGGACACACTCTCCAGTATTGCACAGCAATGCACGCGGTGAGACCCTTTTTCACCTGAGTGGAAAATTAAGTGTGTCAAAGCTGA
GAACTATTGCTGACTTGATGAAAGCTAACATTTTTCGATACCCTCTTGTCCCATGTAAACATAAAATGAGTCAGCCATCCCAGTGGCCACATAGAGATGA
GCCAGATAGTGACAACAGGATTATACAGCTGTCAACAGCTTCCCAACCCCACTTTCAAGAGTGGTTCAGCTCCCCACCTTCTCCTCTGCCCATCACACTC
CTTCTCCAATACAAGCCAGATCAGCGTCTCCACGATGGAAAATTCTCAAACCTAATTCTGGCTTTCCCCAGTCAGATAATGCTACATATCTAGCAGGTAC
CTCTATACCCAGTGTCATCCTAAGCATATTCACGTAACCTCTGAAAAGAAGGAATGGATAACTCCTGTCACCAGTTGGTATAACTACTTGTTCAGTTAAA
TCATCCAGGTCCACCTGTCCTTCCCTTCCTCCAGTCCAGTGAGAAGGGACTTCATAGGACTTCTGGGTTATGAACCTGAAGAGTTATAGGGTTTTGCTTG
GAAAAATCCGGTCACAAAATTAGTTTAGCAGCAACCTGGAAACAAAGTACAGCTTGCCTGTGCCAAGTGGAACCAGTAGCAACATCAACCCTACACCTGA
GCTGGACAGTCACAGGAATATATCAAAGCCAAATCCCCTTCTGAGACTCTCATGTCCTTCTTCTTTCTTGTGGGCAGTCTAAGCTTTGCCTAACCTAAGG
TCTGCCTACCAGTCTCATCAGCCCTCGCAGCAGGTGGCACTGGTGGCTTTGAAGGAGATGCTCTTGTCTGCCCAGMGTAGGTCATGTCACATCACAGAAA
GGACAGAGCGCTGGGACCTGGATTTATAACACACAAGTGCATCCCTGTGTGTCCTTAAAGCAAAGAGTAGCTTGAGCTGCTGTCCCTGAGAGCAGCATCT
GGATCTCACCACAGATATCCCCAAGAGCATTCGGTGGGTTGTAATGAGGACCAGCCAGGTTGCCTCAAAGCTGTGCGTGCACAGCACTGTATTTTGCAGC
AGGGAGCACCTCTCCTTCCACATGGAGGAGAAGAGGAAGAGAAAATGGAAGAGAAAATAAATTATCATCATGCACATGAAGAGGACATCTCATCCCAGAC
AGCATGGGAGACGCTCCTTGGACACTGCGGTCTCAGCTGTGCTCTTGGGGGCCTGACTCTGCTGCTTCCAGTGGCATCCCTTGGGACAAGACTCTGGTAC
TCACCGTGAACTTGACAGTCTGAGCAGCTGTCAATAATCTTACYGTTAGGTTTTGCTTTCCAGATCCCAGGGAAGTTCAAGGAAGCACTTGTCTGCCAGC
AGTGGACTGGGAGTTCTTAAATGAGTATCAGCATTAAGCTGGAAATTCACAACAAGCCTGGATCAAGTTCCTAATCAAGAGGCACATTTGGGACATTTTA
AACCCAAATGCCACCTCTTGGGAGCAGCACAGGCCCTGCACCTCTGCAGAGAGTCACACACTTTGCTCAGGAGCAGGAGAGGAGGCACAGCCCGTCACCC
CCTGGGACACKCAACACGGTGACTGGAGGAGCATGGCACTGCTGGCGGAGTGTCCCAACCCAGATGGCCCAGCTGAACCTGCTGGGCAGCAGAACTCCCC
GGCACTGCCAACTGCACCAGCACCTGCTCCCGTGCTGCCCGAAGCACAGCTGTCTCCTGTCCCCTCTTCTCCTTACTCCCCCAGGAGATTGCACAGCATC

CGTGGTCCCCTGGCCACCAATGCGGCCCTCCCGCCAGGGGTCCCCTCGCCCACTGGCCAGGTCTGTTTGAATCGAGGCATGAGAATTTCTCCCCCATTCG
CGCAATCCTCTTGCCTCCCCCCAGCCATCAAAATCATCCTTCCCTGCCTCCCTCTTTCTCCTTCCCGCCGCCTCCCCCTTCTCTCCCTGGAACTTCCCTG
CAGCTGCAGAGCTGGCCAGACAATCGGGTGTTTTAGAGAAATACATTTTTGGGCTAATTTGCCATGCATAAGTGTTTCTCTTACCCTTTAAAGGCAGGGA
ACAAAGAGCACATTCTCCGGGAGCTCTGCCCTGTTGTGAGCAGCCCTGCAGCTGTACAGCGCTGTGCTACCCTCCGCGCTCCTGCTCCGCTCTCTGCTAG
CTGCACAAGGAGACAGGGCTGAGCTTCTCCCCGAGACAACGCCATCTCCTACCCATCACTACACATTTCTTCCTGGTTTGTTTTGTTTTGTTTTGTTTTT
AAAGTAGAGCCAGAACAGTGGTGAGGGAAGGGATTTGGCAGCACTGTCACCTAGACTGACTTGCCAGATCATCCAGGTACAGTCTTTTGTATTGAGACAT
CATCTATTTGGAAAGTCTGAATTTTTCTTTATTTGGGGCTTAATCCTGTGTCCGGGTTAAGTATTCTATTCCCTCAATAAGTAACACTGTGGCAATGCAT
TACACACACCCACATTAGACTGCATGAAATTTTGTGCCATGAAGGAGCCCTAGTCATTAAAAAAGAAGTCGTGTGCGGGGTTTTCAGGTCCCGTTTCCTC
ACTTAAAGCACTGGTAGTTATCCCTGATTGGGGAATTTCTCAAACACGAGGATTTTCCTTTGTAAGGATAGGATGTTGAATGATGAACAGATTTGACCTG
GTGGACCAGATCAGGAATGAGATACAGATATTCTGGCATGAACAACTCATGAACATGCAAGGTTGTGGGAGAAGATGGCCTGGAGCAGCTGGACACTGAG
GAACTGAAGTTTTTTATCAGCTTTTTGGGGACCTCATTTCAGCACTTGCTGAATAATCTTTTTCCTACTCATGGAAAGCTGGTCCAGCAAATGGGGGCAA
CAAGTTCTGGTGTAAGTGAAACAAGGCTTTTAAAGAAGCCGCAATGAAGTCATTCACCCCATTTCAGCAGGCAGGTCCCACCAAACTCTTCCCACCAGCC
CAATCCCATCCACTGTAAATAATCTTAACTCAGTCCAAACCAGCCACATTTTCAGCTCTGAATTCAACACAATCTAAAGAAGCTAGCCCAGTCAAGTCTA
GCCTATCCAGAGCATGCCTATCACAAGCTACCATCTCAAACATGCATTCAGATTGCACTCAGTCCATGTAAGTGTAGCTCATTTGGACTGAGGCTAGTGT
AACTCAGCCCAACCCAGCCATACCAGGACATTCTGAGACATTTCAAGCCACGCTCCAGTTGGGGACTAGTATAGTCTAGCCTGTCAACTTACACTCAAAG
TCTGACCCAGTAACGTGATGAACCAGGATGTGCCAGCTGCAAGGCAACAGAAGCTCAGTTTTTACACACTTGAAATGTGTTTTTAGGTTTGAAAATGTCT
TACCCAGAGTACAGAGATCAGCATGGCTTTCAGAGTTCCAGCTTGTGGGACCTTGTCTCAGATAGGTTAACAGCATTGGTCCATTTGAAATGCTCTTCAC
ATCATACTGATACAAATACACTTGTTCTTCAGTTCTCTCCAGCACATTGCACCCTATAATTTTGCACCCAGAAGATCCTCTTGACCCAAATCCAGATGGA
TCTCTTGCAAATACAGAGAAAGATAGAGACTTTGTTTTAAAATTCTTCTATCTAAGAAGAGAAGGGAAAGGAGGAGAGGAGAGGAGAGGAGAGGAGAGGA
GAGGAGAGAGGAGAGGAGAGGAGAGGAGAGGAGAGGAGGAGGACTGCAGTCAGCAGAAATGTATTTGGAGAAGCACATAAAGCAGGAAAAGGAGAAGGTC
TCACGAACTGAAGAGCAAAATCCTAAGCCTCTCAGCTAGGAAAGGCCAGCTTGGAGGAAACAATAGCTGGAGGAATGTACAGTAGAAGATGGTGTAATTG
GCAACAGCACCCCAATGCTAGGACTGTGGGGCAGGTTGAGCCTCACCTGGCACAGCCCACAAAACCCTTCAGTTCCTTTCTGGCTTTTTGAGCTCTTTGC
ACTCAGTTCTTCAGTAGCCATACACATGTAATAACTTGGTTACAGTAGGAAGCCATTAGTGGAGCTGAGGCTTAAAGACGTGTGTTTATTGACAGTGTGG
TGGCACTTGTTCCACACAGAGCACCACCAGCTCTTAGCTCAGCTGTGGTCTTACTGCTCTGAGACTCCTGCTTTGCTATGCTACTGCGAATGAGTTGTAT
GAGGAAGCAGTGGAGAAATTCAAGATTCTTAGTTTCCAAGCTGCATTTTAAACTCCAGAATTCACTCTTTTGACCTTACAAGTGACTCCAGTAAGCGCCA
GTCAAGTATTAAAACCGTAATGCCAAGAAAAAGTCAATTTCTCCTGGACTCAGAGCTGCAATAGTGAAAATCCCATTGAGCAATGGGTTTAATAATATTT
AGCATCCCCTGTGGGTAAATGTTCTTCGATCCCTCTAGGAGACTGGACTCGAACATTTCCCTGGCAGGGCTGTAGGGGACATCACACAGCACAGGTGGTC
TGAGTGGCACAGGTAGGTTGGACGCCAAGACCAAGTGCCAACACAAACACTCTGAAGCCCAGGATGAGGTTAGCAGGACCAAAATCAGCTCCAGCCTTTG
TGGTTACCATCTCAGTAAGATAGAAAGAGAGATAGAAAGGAGACAGAAGGGAGTGAGAAGGTTATGGAGTGGGGGAGGGCTGGGAGCCTGCAGACTTCTG
TTGCCACATGATTTTCCTGATGTTTTCAAAGTGCTGCTCTAACACACGCGCATGAGCCCCATGTGACAGCTTATTTACGAAAGCACAGGCGTAATGTTGG
GTACATCTCGCATCCGCTCAGCAGCCCACCTGACTGCCAGCTCTGTGTGCACCCACTGTGTCCTCCCCATCTGCGCCATCGAGCCAAGGGGGATGGGTGA
CCCATGATGGGTGGACATTGCTCCTGTCTTGGCAGGGKGGGGAAGCGAAGAGAAGGACTGTCTTTCCATTTCCAAACTGCTCTTTACTTCTCTTTGCCTG
GCACTACATGAGGAGACTCCAGACGCAGGATGCTGTCCCTTCTGTAAGAGAAAAAGCTTTTATTTCCGATCTGGCTGTCTCACTTCTTTTAATTTGCAGA
GCACCTGGAAGCAAGCTCAGATACAAACTGATTCAGCGTATGCCAATATCCCATACTTGATCTTCAAACAGATGCAGATTCTCTTTCCTTGGATATAAAT
TTTTCTTGTACCATCATCACAGAAGTTCCATTTCACCCAAAGATCCACTCAACAAAAGAGGAAGCCTCCTTAGAAAGGTCAGGCAGAAGGCTAAGGGAGA
GAGTTCACAATGAAATAAAGCTGGTTGTTTCTTCAGGGATGGAGGATTTGGCCTACCTTCAATTCATTTGCATACAGAACCCCTAGCTCCACTAAAATTG
TCCATCTCTGCCAAAAGGAGGTCTAACACATGAGCAAGAGAGATGCAGTCTGCGAAAATGGAGTTTCTGAAGGACAGCAATAGGAACTGAGTTCACAGAG
GCTGTTAKGGGACAAGATTATGCTGCCATAAAGCATATATTTCAGGAATGGCAACTTCCTTTCCTTTCTCTCCCCATAGTCCTAGGTGCTAGCATTCCTA
GAAAAAATGTCAGTGGAAAAAAGTCATCACCTCATATGCTGAAAAAAAAACACAACAGTGACCTTTGCCAAAACAGAAGCTCCTATGAAGAAAGGATATC
TGACATTTTAAAACTTTGATATGGTCAAACCACACAATGTCATCCCTACCTCCTCTTTCCAGACAATCAGTCCTTGCTTTTAATGACCCAAGAGCCAAGG
GCGCTACCTCACCACACAGATCACGCCTCCCATGGCCATGCCTCTTTCCTGCACCTCAGTAAGTTGCTTCTAGGAAGGGTTAATTCAACCAGGGCCTTTT
ACTCCAAGTTGTTTTCCAAGTAGCTGAGAGAATAGGTCTCTTGTTACAAACATGGCTTCTGGGCATTTGGTGATGCTTTCCCTTTTCTCTCTCTCTGTAC
CACAAAAGAACCTTTTGTTCTTGTTCCTCTGTCTCATTTACTCTCCCCCAGCCTGCTCTCTCCTCTTCCCTCCATTGGCTGCCAAGKGACTAAGGAATGG
CAAAAAATGACCATGGCGCGGTCATGGCAAATATTTCTTCAGCTATTATCTGATAGAGACCTTGAGCATCTACGCGAAAGGTCCTCTGCCTTCCCTCAGG
CTTCTCCTAGAGATGGGGAAAAATGAGTCAGACACAACATGGCAGGATATAAGTTGAATCAGCCCAACATGCTAATGGGAATGTCTTGCGAATGGAGTCA
TGGTGCCTGTCTCAGGGAAATCAAATGATAGGGACCCAGAACTGGCCGGAGTCCAGTGGCAGATTCAATGGGTCTAGATTTTTATAGGAACTCTCAATTT
ATTAAAAAGCACACAGTAGGCACGAAAAGTCATTTTCTTGCCTCCACATTTACTGATTCAGCTGTTTCTAAGCTGGAGCTGACACTCTTTGTCAGATGGG
TTGTAATATTGTATAGTTTCTCCACACCATTTATGTAAATAAATTGTGCATGATCACCCACCACAGCAAAGCTGTGGCAAATCCCCATGGATATCACGTC
TTCCCACACCCCACTGAAATTACACCTCAGGCATTCCCACATACATACTTCTAGCACGTGCGGCTTCACTGAGTCATTTTACACGTACATACATAGATAC
ACAAAAGTTCACATGACGGTAAAGTCACCGAAACTTCCTTGGTGACCTTTCTGCAACAGAAACAATTCCCCAGACAAATTCCCACCTCCCTACCTTTTAG
AGCATATGGGTATATCCTTCACTGATACTGCCTTCTGAAAAAAAAAAAACAAACTAAAATTCTTCAGTCTCACCTGAAGCAAATCTTGCTGTTTCTGCCT
TCTCATCTTCTTCTTTACTGACCCTCTTCACTCCTCTGACTACCCTTTGTCCCGAGTCCATCTCCGTCCATGTTGTGCTTTAGGTGAACATGGCATTTGA
CGGCTGGGTCCATCTGCCAAGCTTTTTGCACTCTTCTCCCATTCAGTGCCTTAGCTATTTTGGAAGGCCCTTGAAAAAATCATTAAATTTCAGCCTTAGT
GACTTCTCGCACACACGCATGCAAAGCTGTGGATATAAGGCGTTATCTGGAGAATTTTTATTCCTGTAGAGAAGGGGAATGGACTATACAGATAAAAGGG
CCATTATATTCCCACATTAGAAGGACTTCTTGTCTGGTGGAT
