# Supplementary material for: A DNA Replication Mechanism Can Explain Structural Variation at the Pigeon Recessive Red Locus
Source: Biomolecules. 2022 Oct 18;12(10):1509. doi: 10.3390/biom12101509 (PMC9599118; doi:10.3390/biom12101509)
Supplement: Supplementary file 1 [file biomolecules-12-01509-s001.zip › Supplemental Figure S4.pdf]

Figure S4. ***e***^{2} **allele annotated relative to** ***E***$^+$

Annotation Key

Left side of the deletion

Right side of the deletion

SINE/MIR

simple repeats

low complexity GA rich

enhancer MCS4

enhancer MCS5

Sanger-sequenced gap in reference genome

GTGACCCAAAGCACTGTTGTCAGCCCTACCATATGGACTATGAGGTCTTCAGAACAACTCTCTGATCAGCCCTAGGTGCGTACATGGCTGCAGCACTGAC
TAAGAGCTGAGCTCAGCAGCCTGACAGTGAGTGCAGGTTGGGCAGACGGTGACCTGCCTGAAATGTGAACAGAGCTGGTCTGGAGCACACAAATTGCTTC
TGTTGGCCAAAACACATGTCACCAAAGCATTTCCTCCTCCCCACACACACCAGGGTGGTGCAAAAACAGGCTTGAGCACTATCTCTGTACCTTAGCATCC
TCTGAGAGATGCGCGATGCCACCAGCATGSSAYTCCTCCACCCCCTCCCCGCTCCCTGTACCACCACAATGAGCATGCCTTGTCGTAGAGGGTTAATAAA
TGATGCAAGAAAGGAGCCTTTTAATGCATGGTGACCTTTGACCTTTCATAAATCACAGTGTTGGGCTGTGAGTGCGCGGGGGGCGCAAGGCAGTGGGGGG
AGAGGAATGGGAGGGGGTCCATGCAGCTGCGCCACCGAGTTGACATTGTTCCCACCATCACTAAAGTGCAACAAATCCCTCTATTGTGTTCCTGGTTTAT
CTGGTTCCTCTTGTTTATTAGCAGAGGTTGTTTGGCGCTGGCTCCTGAGCCCTGGGCGGGTGGAAAGAGTGTTGGCACGCACCAGAGGAGGAGAGGGAGGGA
GTGGGGGGGACCGGGACGGGAGGACGAGAGCAGTTCATCAATGGCTGTATTGTCCCATCTTTTTTGTTGCTCCTGTAATGATATGTTCGAAAAAAGGGGA
TTARAAAAAAAAAAAGGCAGGGGGAGACAAGAGAGGGAGAGAAAGACAAACCACATAGAAGGACTTGGTAAGAACGGCCGGTCTGGGCCCGCAGCGGATG
CTGTCGCAGCTGGGGAGGATGGGTCGGGACGGAGCCGCGACGGGGCTGCTCTGTGCCTTTATGCTGCTTATTCAGGTCCAGATTCGTGAGCCCCAGTGGC
TCAGTCAGGCTGGGCCCACCCGTGCCAACTCTATTAATGACCAGTGGGGACCATGTGCCCATGGTGGAGAGGATGGGGTGGCAGGAGCGAAGGGCTGGGG
CTGGTGGGGAAGGGAGAGGAGGGCAATTCTTCACATCCATCAAGTCTGAACCTGCCTTTGTACAGAAGAGGCAGTTGGGGGGCACAGCACCTTCTCAAAG
CACTGGCGGTGGACCAGATGATCCCTGTGGTCCCTTCCAATCTGGTATGCTATGCTTCTATGATTCATCCCTCTTGGTCATTTTTCCCCAACCTGCTCCC
TCATTATGAGAAGCAACAGGKYCCCTTCCTAAATTAGCAGAGATTTTTCTTGTGCCTGGGCCTTACTTACACGAGCATTTTTCCCCCTGGAGCTGCTGAC
TGCCCATGGAGAGCTCCTCTGGTTTTCATAGGGATCGGCAGGACAGTGCTGGGAAATGATGGCACTTTGGTTGCCGCTTTACCCAAAGCTAGATGATGAT
GTGAATAAAACACCAGGGCCACAGGGACTTTGTTCATCATTGCTCTGCCTTAGCTACCCATGGAGCTGGACTGAAGCAAGCATCCTAAGGGGTGCACGTG
CCACCATAAATTGCCTTGTTGAGATCAAAGTGAGATTTATTGAGAGAAGAGTGAAATTCGGTATGTGATGTTCAAGGGCATTTTCTAGTTCATCTCAGCC
CACCATATAGCTGTTCTTTTCCCAACCACACCAGCCGTTTCTGCTCCAGTGTCTCCATTACCTCAGAGGTGTCGCATAGGGGCACATTGATCTGGACATG
CACAGGTGCTCTGCTGTGATTTATTTTTTTTCTCCAAATGGTATAATTCAGAGATTAAAACATTCTGCTGAACTTGAAATAGCAGGCTTTATTTTGTGGT
TATTAGGCTCTTGTGCCTGACCTCTCATCTTTCAACCTGGGTCAAGTTTTAAATGCAAAATGCTGGTTTGAAATGAAATGTCAGAAATTCCTTTGAAAAT
GTCCATTCGCAACATTTTGGCATCTAGAAAACAGAATAATTCAGGAAGATTGCATTGAAATGAAATGTTTTTGATATTTTAAAAATCCCCCTCCCACACT
CACTTTCCAGCCAGAATTTCTTGTTGAATTAGGTCTGGATCTGCAACTGGTTTCCTTCCCCTAAGTCCCATTTCTTGGCAAATTATTTACCAATAAATGC
TTCCCTAGTACCAGTCTTTCCTACCCTCAGATGATGCCACATCCTTGCCATCCCTGAGAGGTCTCAGGGTCTCCACAAAGCACTGGAGTGTGTAGGAGAA
GTGGAGAGGAGGGGATGGGACACACTCTCCAGTATTGCACAGCAATGCACGCGGTGAGACCCTTTTTCACCTGAGTGGAAAATTAAGTGTGTCAAAGCTG
AGAACTATTGCTGACTTGATGAAAGCTAACATTTTTCGATACCCTCTTGTCCCATGTAAACATAAAATGAGTCAGCCATCCCACTGGCCACATAGAGATG
AGCCAGATAGTGACAACAGGATTATACAGCTGTCAACAGCTTCCCAACCCCACTTTCAAGAGTGGTTCAGCTCCCCAGCTTCTCCTCTGCCCATCACACT
CCTTCTCCAATACAAGCCAGATCAGCGTCTCCACGATGGAAAATTCTCAAACCTAATTCTGGCTTTCCCCAGTCAGATAATGCTACATATCTAGCAGGTA
CCTCTATACCCAGTGTCATCCTAAGCATATTCACGTAACCTCTGAAAAGAASGAATGGATAACTCCTGTCACCAGTTGGTATAACTACTTGTTCAGTTAA
ATCATCCAGGTCCACCTGTCCTTCCCTTCCTCCAGTCCAGTGAGAAGGGACTTCATAGACTTCTGGGTTATGAACCTGAAGAGTTATAGGGTTTTGCTTG
GAAAAATCCGGTCACAAAATTAGTTTAGCAGCAACCTGGAAACAAAGTACAGCTTGCCTGTGCCAAGTGGAACCAGTAGCAACATCAACCCTACACCTGA
GCTGGACAGTCACAGGAATATATCAAAGCCAAATCCCCTTCTGAGACTCTCATGTCCTTCTTCTTTCTTGTGGGCAGTCTAAGCTTTGCCTAGCCTAAGT
CTGCCTACCAGTCTCATCAGCCCTCGCAGCAGGTGGCACTGGTGGCTTTGAAGGAGATGCTCTTGTCTGCCCAGAGTAGGTCATGTCACATCACAGAAAG
GACAGAGCGCTGGGACCTGGATTTATAACACACAAGTGCATCCCTGTGTGTCCTTAAAGCAAAGAGTAGCTTGAGCTGCTGTCCCTGAGAGCAGCATCTG
GATCTCACCACAGATATCCCCAAGAGCATTCGGTGGGTTGTAATGAGGACCAGCCAGGTTGCCTCAAAGCTGTGCATGCACAGCACTGTATTTTGCAGCA
GGGAGCACCTCTCCTTCCACATGGAGGAGAAGAGGAAGAGAAAATGGAAGAGAAAATAAATTATCATCATGCACATGAAGAGGACATCTTATCCCAGACA
GCATGGGAGACGTTCCTTGGACACTGAGGTCTCAGCTGTGCTCTTGGGGGCCTGACTCTGCTTCTTTCAGTGGCATCCCTTGGGACAAGACTCTGGTACT
CACCGTGAACTTGACAGTCTGAGCAGCTGTCAATAATCTTACCATTAGGTTTTGCTTTCCAGATCCCAGGGAAGTTCAAGGAAGCACTTGTCTGCCAGCA
GTGGACTGGGAGTTCTTAAATGAGTATCAGCATTAAGCTGGAAATTCACAACAAGCCTGGATCAAGTTCCTAATCAAGAGGTGCATTTGGGACATTTTAA
ACCCAAATGCCACCTCCTGGGAGCAGCACAGGCCCTGCACCTCTGCAGAGAGTCACACACTTTGCTCAGGAGCAGGAGAGGAGGCACAGSCCGTCGCCCC
TGGGACACGCGACACGGTGACTGGAGGAGCATGGCACTGCTGGCGGAGGGTCCCAACCCAGATGGCCCAGCTGAACCTGCTGGGCAGCAGAACTCCCCGG
CACTGCCAACTGCACCAGCACCTGCTCCCGTGCTGCCCGAAGCACAGCTGTCTCCTGTCCCCTCTTCTCCTTACTCCCCCAGGAGATTGCACAGCATCCG
TGGTCCCCTGGCCACCAATGCGGCCCTCCCGCCAGGGGCYCCCTCGCCCACTGGCCAGGTCTGTTTGAATCGAGGCATGAGAATTTCTCCCCCATTCGCA
CAATCCTCTTGCCTCCCCCCAGCCATCAAAATCATCCTTCCCTGCCTCCCTCTTTCTCCTTCCCGCCGCCTCCCCCTTCTCTCCCTGGAACTTCCCTGCA
GCTGCAGAGCTGGCCAGACAATCGGGTGTTTTAGAGAAATACATTTTTGGGCTAATTTGCCATGCATAAGTGTTTCTCTTACCCTTTAAAGGCAGGGAAC
AAAGAGCACATTCTCCGGGAGCTCTGCCCTGTTGTGAGCAGCCCTGCAGCTGTACAGCGCTGTGCTACCCTCCGCGCTCCTGCTCCGCTCTCTGCTAGCT
GCACAAGGAGACAGGGCTGAGCTTCTCCCCGAGACAACGCCATCTCCTACCCATCACTACACATTTCTTCCTGGTTTGTTTTGTTTTGTTTTGTTTTTAA
ACTAGAGCCAGAACAGTGGTGAGGGAAGGGATTTGGCAGCACTGTCACCTAGACTGACTTGCCAGATCATCCAGGTACAGTCTTTTGTATTGAGACATCA
TTTATTTGGAAAGTCTGAATTTTTCATTATTTGGGGCTTAATCCTGTGTCCAGGTTAAGTATTCTATTCCCTCAATAAGTAACACTGTGGCAATGCATTA
CACACACCCACATTAGACTGCATGAAATTTTGTGCCATGAAGGAGCCCTAGTCATTAAAAAAGAAGTCGTGTGCGGGGTTTTCAGGTCCCGTTTCCTCAC
TTAAAGCACTGGTAGTTATCCCTGATTGGGGAATTTCTCAAACACGAGGATTTTCCTTTGTAAGGATAGGATGTTGAATGATGAACAGATTTGACCTGGT
GGACCAGATCAGGAATGAGATACAGATATTCTGGCATGAACAACTCATGAACATGCAAGGTTGTGGGAGAAGATGGCCTGGAGCAGCTGGACACTGAGGA
ACTGAAGTTTTTTATCAGCTTTTTGGGGACCTCATTTCAGCACTTGCTGAATAATCTTTTTCCTACTCATGGAAAGCTGGTCCAGCAAATGGGGGCAACA
AGTTCTGGTGTAAGTGAAACAAGGCTTTTAAAGAAGCCGCAATGAAGTCATTCACCCCATTTCAGCAGGCAGGTCCCACCAAACTCTTCCCACCAGCCCA
ATCCCATCCACTGTAAATAATCTTAACTCAGTCCAAACCAGCCACATTTTCAGCTCTGAATTCAACACAATCTAAAGAAGCTAGCCCAGTCAAGTCAGCC
TATCCAGAGCATGCCTATCACAAGCTACCATCTCAAACATGCATTCAGATTGCACTCAGTCCATGTAAGTGTAGCTCATTTGGACTGAGGCTAGTGTAAC
TCAGCCCAACCCAGCCATACCAGGACATTCTGAGACATTTCAAGCCACGCTCCAGTTGGGGACTAGTATAGTCTAGCCTGTCAACTTACACTCAAAGTCT
GACCCAGTAACGTGATGAACCAGGATGTGCCAGCTGCAAGGCAACAGAAGCTCAGTTTTTACACACTTGAAATGTGTTTTTAGGTTTGAAAATGTCTTAC
CCAGAGTACAGAGATCAGCATGGCTTTCAGAGTTCCAGCTTGTGGGACCTTGTCTCAGATAGGTTAACAGCATTGGTCCATTTGAAATGCTCTTCACATC
ATACTGATACAAATACACTTGTTCTTCAGTTCTCTCCAGCACATTGCACCCTATAATTTTGCACCCAGAAGATCCTCTTGACCCAAATCCAGATGGATCT

CTTGCAAATACAGAGAAAGATAGAGACTTTGTTTTAAAATTCTTCTATCTAAGAAGAGAAGGGAAAGGAGGAGAGGAGATGGAGAGGAGAGGAGAGGAGC
TGTTTCTAAGCTGGAGCTGACACTCTTTGTCAGATGGGTTGTAATATTGTACAGTTTCTCCACACCATTTATGTAAATAAATTGTGCATGATCACCCACC
ACAGCAAAGCTGTGGCAAATCCCCATGGATATCACGTCTTCCCACACCCCACTGAAATTACACCTCAGGCATTCCCACATACATACTTCTAGCACGTACG
GCTTCACTGAGTCACTTTACACGTACATACATAGATACACAAAAGTTCACATGACGGTAAAGTCACCGAAACTTCCTTGATGACCTTTCTGCAACAGAAA
CAATTCCCCAGACAAATTCCCACCTCCCTACCTTTTAGAGCATATGGGTATATCCTTCACTGATACTGCCTTCTGAAAAAAATAAAAACCAAACTAAAAT
TCTTCAGTCTCACCTGAAGCAAATCTTGCTGTTTCTGCCTTCTCATCTTCTTCTTTACTGACCCTCTTCACTCCTCTGACTACCCTTTGTCCCGAGTCCA
TCTCCGTCCATGTTGTGCTTTAGGTGAACACAGCATTTGACGGCTGGGTCCATCTGCCAAGCTTTTTGCACTCTTCTCCCATTCAGTGCCTTAGCTATTT
TGGAAGGCCCTTGAAAAAATCATTAAATTTCAGCCTTAGTGACTTCTCGCACACACGCATGCAAAGCTGTGGATATAAGGCGTTATCTGGAGAATTTTTA
TTCCTGTAGAGAAGGGGAATGGACTATACAGATAAAAGGGCCATTATATTCCCACATTAGAAGGACTTCTTGTCTGGTGGAT
